# The Acid Roles of PtSn@Al_2_O_3_ in the Synthesis and Performance of Propane Dehydrogenation

**DOI:** 10.3390/molecules29132959

**Published:** 2024-06-21

**Authors:** Hejingying Niu, Jinhua Ma, Lina Gan, Kezhi Li

**Affiliations:** 1 School of Environmental & Chemical Engineering, Shanghai University, Shanghai 200444, China; niuhjy@shu.edu.cn (H.N.); mjh21722719@shu.edu.cn (J.M.); 2 School of Environment and Architecture, University of Shanghai for Science and Technology, Shanghai 200093, China; 3 Institute of Engineering Technology, Sinopec Catalyst Co., Ltd., Beijing 101111, China

**Keywords:** PDH, PtSn/Al_2_O_3_, citric acid, thermodynamic analysis

## Abstract

In this study, a PtSn/Al_2_O_3_ catalyst with bimetallic uniform distribution in the sphere was synthesized. The PDH performance and characterization analyses, such as with FTIR, XPS, and NH_3_-TPD, were investigated. The effects of acid on the PDH performance were analyzed. Citric acid (CA) acted as a competing adsorbent in the preparation process of the PtSn/Al_2_O_3_ catalyst to synthesize the uniform catalyst. Water washing and alkali-treated samples were also studied. SEM line scanning revealed that increased the apparent concentration of Pt metal from 0.23 to 0.30 with citric acid. In contrast to the fresh PtSn/Al_2_O_3_ catalyst, the addition of citric acid increased the PDH selectivity from 74% to 93%. After alkali or water washing treatments, the catalyst’s selectivity further increased to 96%. Strong acid sites promoted the breaking of C–C bonds during the PDH reaction, resulting in more methane and ethylene byproducts, and decreased catalyst selectivity for fresh PtSn/Al_2_O_3_. From the PDH reaction thermodynamic analysis, a relatively sub-atmospheric pressure environment with a lower propane pressure could be the reasonable choice.

## 1. Introduction

Propylene, as one of the fundamental raw materials among the three major synthetic materials, is used to produce industrial products such as propionaldehyde, polypropylene, acetone, acrylonitrile, and epoxy propane [1,2]. Recently, with the development of economies, the demand for propylene has steadily increased. However, as fossil energy resources are rapidly depleting, propylene production through traditional processes is unsustainable. Therefore, some emerging propylene production processes have gradually evolved [3,4,5,6,7]. Propane dehydrogenation (PDH) technology, with simpler reactants, limitations in by-products, and lower investment costs, is one of the most promising methods for olefin production. In the PDH industry, platinum-based catalysts are widely employed. However, the low selectivity for propylene reduces the propylene yield [8]. From previous studies, the structural characteristics of carrier materials, such as the specific surface area, acid–base properties, and thermal stability, can affect the catalytic performance of Pt-based catalysts in PDH processes [9,10,11,12]. Therefore, amounts of researchers have attempted to regulate the acidity and alkalinity of catalysts to improve their performance and increase the propane conversion and propylene selectivity. Ponomaryov et al. [13] introduced NaCl into MFI zeolite using impregnation, which significantly improved the distribution of Pt, effectively suppressed the acidity of the zeolite, and prevented the sintering of metal Pt during the calcination process. Jang et al. [14] controlled the acidity and alkalinity of a γ-Al_2_O_3_ support by varying the calcination temperature of the carrier, revealing the relationship between Lewis acid sites on the carrier surface and coke behavior. The study showed that the deactivation rate of the catalyst significantly decreased and the stability greatly improved during the PDH reaction process after high-temperature calcination. Due to the fact that high-temperature calcination reduces the total amount of acid sites on the γ-Al_2_O_3_ support, the result is a decrease in the amount of coke produced during the reaction. On the other hand, scholars have studied the role of acid sites in PDH reactions [15,16,17], and there is still no systematic explanation for which acid sites play a key role in improving the selectivity of catalysts. Based on this important issue, a strong theoretical guidance for the development of efficient catalysts needs to be discussed.

To elucidate the role of acidity in catalyst synthesis and performance, we employed the impregnation method to prepare PtSn bimetallic catalysts with γ-Al_2_O_3_ as the support. The impregnation depth was controlled using citric acid as a competing adsorbent during the impregnation process. The PtSn/Al_2_O_3_ was also treated with alkaline treatment or water washing. Basic characterizations of the catalyst, including the morphology and chemical state of the active metals, were conducted using SEM, XRD, BET, XPS, and other methods. The Pt species in the catalyst samples were investigated with CO-DRIFT, and the acid sites of the catalyst were characterized using NH_3_-TPD. This exploration aimed to further study the influence of the support’s acidity and basicity on the catalytic performance of the catalyst. The results indicated that after adding citric acid for regulation, the strong acid sites were significantly reduced, and the by-products, such as methane and ethylene, caused by C–C bond cleavage were reduced. The isolated Pt sites were increased, and the selectivity of the catalyst was greatly improved.

## 2. Results

### 2.1. Dispersed PtSn Catalyst

**Figure 1:**
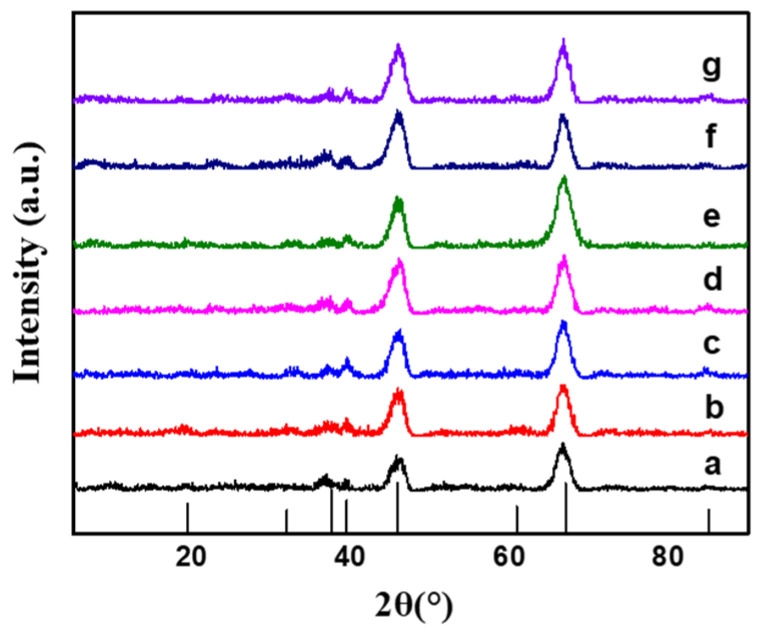
XRD spectra of γ-Al_2_O_3_ support and PtSn/Al_2_O_3_ catalyst: (**a**) γ-Al_2_O_3_, (**b**) PtSn/Al_2_O_3_, (**c**) PtSn/Al_2_O_3_-0.4CA, (**d**) PtSn/Al_2_O_3_-0.8CA, (**e**) PtSn/Al_2_O_3_-1.2CA, (**f**) PtSn/Al_2_O_3_-WS, (**g**) PtSn/Al_2_O_3_-OH.

Figure 1 presented the XRD spectra of the γ-Al_2_O_3_ support and the PtSn/Al_2_O_3_ catalyst, with diffraction peaks at 2θ = 46.3° and 66.8° attributed to γ-Al_2_O_3_ [18]. No diffraction peaks corresponding to metallic Pt and Sn were observed, indicating well-dispersed Pt and Sn on the support surface. Figures S1 and S2 showed SEM images of the γ-Al_2_O_3_ support and PtSn/Al_2_O_3_ catalyst. The PtSn/Al_2_O_3_ catalyst and γ-Al_2_O_3_ support demonstrated a typical type IV isotherm (Figure S4). The isotherm suggested interconnected and irregularly arranged pores, categorizable as mesoporous structures with H_2_ adsorption hysteresis. Additionally, from the pore size distribution of the PtSn bimetallic catalyst and support, the pore size of the loaded metal remains basically unchanged regardless of the pretreatment (Table S1).

### 2.2. Citric Acid Regulates Immersion Depth

**Figure 2:**
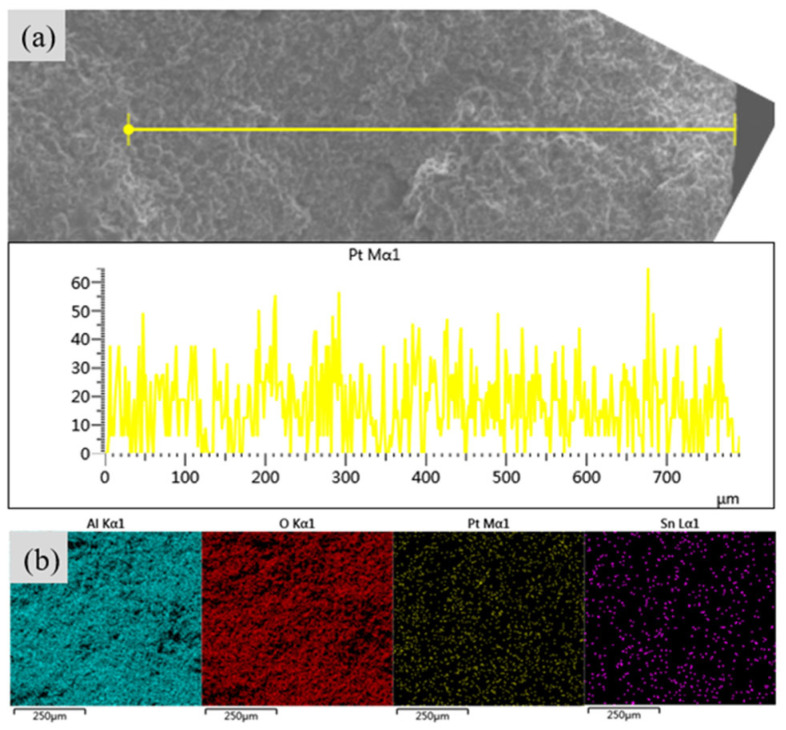
The impregnation of Pt before and after adding the citric acid line scanning of SEM: (**a**) PtSn/Al_2_O_3_-1.2CA and (**b**) surface scanning from EDS.

Citric acid was used as a competitive adsorbent to regulate the immersion depth of active metal Pt. The SEM line-scan spectrum showed the radial concentration changes of the Pt element in the PtSn/Al_2_O_3_ spherical catalyst (Figure 2). It indicates that after adding citric acid, the radial distribution of the Pt element in the catalyst is relatively uniform. By contrast, the Pt element was enriched on the surface of PtSn/Al_2_O_3_ compared to its inside (Figure S3). The quantitative analysis in Table S2 showed the apparent concentration and atomic percentage of the Pt increase when adding citric acid. However, as the citric acid content increases, the apparent concentration and atomic percentage of Pt begin to decrease. In a previous study, hydrochloric acid was used to regulate the immersion depth of Pt and Re on γ-Al_2_O_3_ nanosheets [19]. In an acidic solution, the surface of alumina was hydrated and dissolved by Al(OH)^2+^ ions, and then, aluminum cations and chloroplatinate anions are re-deposited on the alumina. After adding HCl, the dispersion of the active metal is increased by three times [20]. From EDS images (Figure 2b), the active metals Pt and Sn were equally evenly dispersed on the cross section of the carrier. This result suggested that citric acid could effectively change the dispersion state of Pt inside the carrier as a competitive adsorbent. Moreover, under the same pre-impregnation concentration and time, the adsorption rate of citric acid was faster.

**Figure 3:**
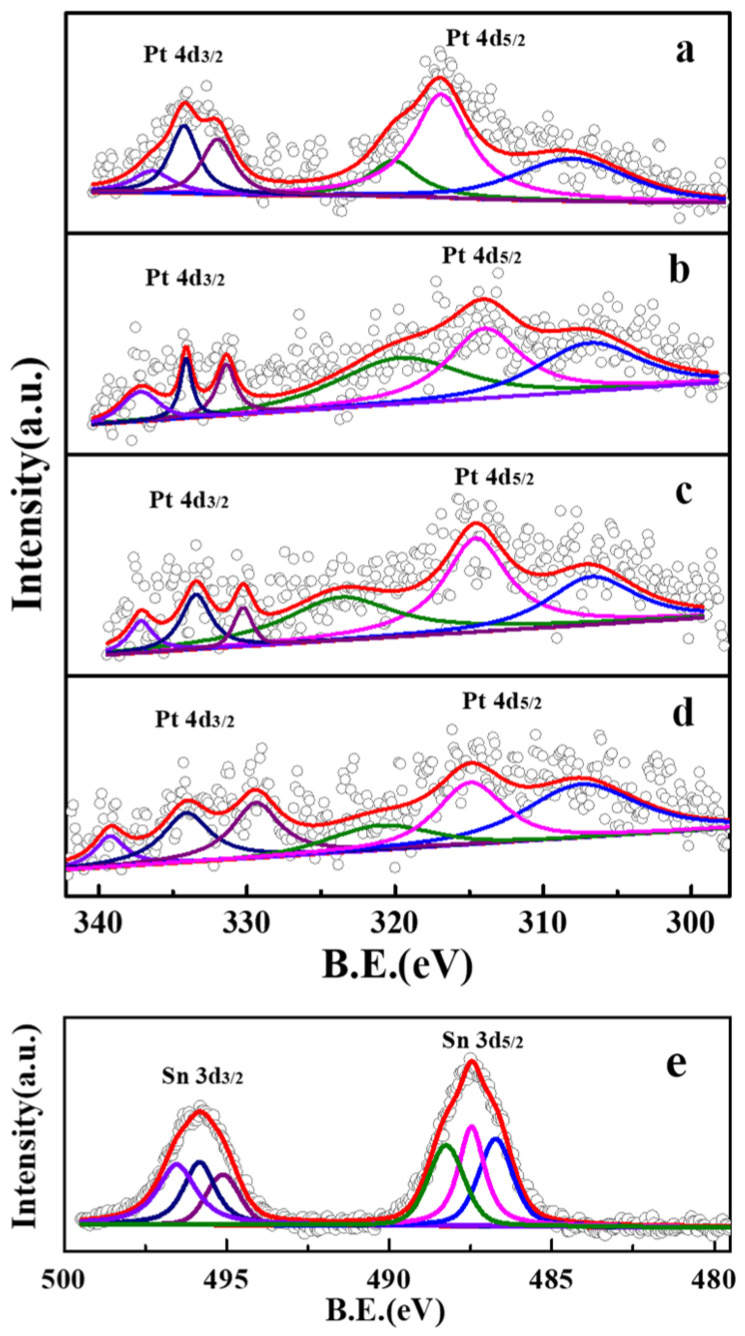
XPS spectra of PtSn/Al_2_O_3_ catalyst with added citric acid and un-calcined Sn/Al_2_O_3_ Pt: (**a**) PtSn/Al_2_O_3_, (**b**) PtSn/Al_2_O_3_-0.4CA, (**c**) PtSn/Al_2_O_3_-0.8CA, (**d**) PtSn/Al_2_O_3_-1.2CA, Sn: (**e**) Sn/Al_2_O_3_.

To investigate the chemical states of the active metals in the catalyst, the XPS test was conducted (Figure 3 and Table 1). The PtSn/Al_2_O_3_ catalyst exhibited Pt 4d peaks at 332.0 eV and 309.5 eV, corresponding to Pt 4d_3/2_ and Pt 4d_5/2_ of metallic Pt, respectively [21,22,23]. Peaks at 334.2 eV and 316.9 eV as well as 336.4 eV and 320.1 eV corresponded to Pt^2+^ and Pt^4+^ [21,22,23], respectively. For the PtSn/Al_2_O_3_-CA catalyst, the binding energies of Pt^0^ and Pt^2+^ shifted to lower energy levels with the addition of CA. It indicated more electron-rich Pt. The binding energy Pt^4+^ in the PtSn/Al_2_O_3_-1.2CA catalyst was the highest, indicating that Pt was in a high oxidation state [24]. However, no signal peak for Sn was detected in the XPS spectra of the calcined PtSn/Al_2_O_3_ catalyst. Therefore, Sn was loaded directly on the γ-Al_2_O_3_ support and detected with the XPS spectrum of the as-loaded Sn/Al_2_O_3_ catalyst. As shown in Figure 3, by fitting the peaks into three types of peaks in the Sn 3d XPS spectrum of Sn/Al_2_O_3_, which belong to Sn^0^, Sn^2+^, and Sn^4+^ species, metallic tin was confirmed [25]. For oxidized Sn species (Sn^2+^ and Sn^4+^), the peaks for Sn 3d_5/2_ were located at 485.8 eV and 484.9 eV, while Sn 3d_3/2_ was at 494.2 eV and 493.2 eV [25,26,27]. Therefore, the active Sn species may enter the bulk phase from the catalyst’s surface.

**Figure 4:**
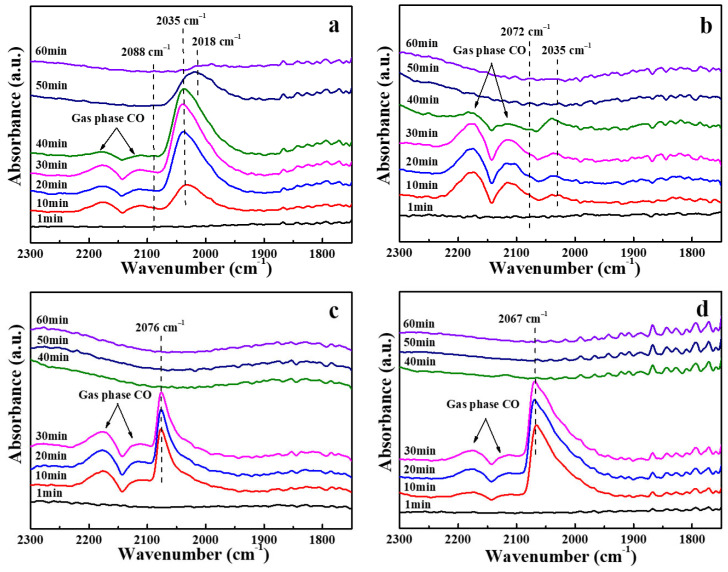
CO-DRIFT spectroscopy of PtSn/Al_2_O_3_ catalyst at 250 °C: (**a**) PtSn/Al_2_O_3_, (**b**) PtSn/Al_2_O_3_-0.4CA, (**c**) PtSn/Al_2_O_3_-0.8CA, (**d**) PtSn/Al_2_O_3_-1.2CA.

The Pt species on the catalyst sample were accurately characterized by CO-DRIFT. As shown in Figure 4a, the weak band at 2088 cm^−1^ was attributed to the linearly bound CO located on individual Pt^δ+^ atoms. The peak demonstrated a red shift direction with the addition of CA until invisible [28,29]. The band 2035cm^−1^ represents Pt^0^ atoms, which are CO linearly adsorbed by isolated Pt nanoparticles [30,31]. This band intensity was weak on PtSn/Al_2_O_3_-0.4CA catalysts but more pronounced on PtSn/Al_2_O_3_-0.8CA (blue shift to 2076 cm^−1^) and PtSn/Al_2_O_3_-1.2CA (blue shift to 2067 cm^−1^) with increasing citric acid concentration. In general, with the increasing positive charge of a metal ion, the contribution of the π bonding to the interaction of CO with the cation decreases, whereas the electrostatic interaction and σ donation increase [32]. The intensity of the band range (2067–2076 cm^−1^) in Figure 4 gradually increased. It indicated that the intensity of isolated Pt sites (Pt_1_) increased. This is attributed to the competitive adsorption effect of citric acid, which makes Pt dispersion uniform, and platinum atoms replace platinum clusters. Previous studies have shown that the Pt_1_ site promotes the selectivity of the catalyst [33]. There are currently other opinions on the impact of the Pt_1_ site on selectivity. DFT calculations indicate that the activation energy of the C–H bond and the dehydrogenation energy barrier of β-H at the Pt_1_ site are smaller during propane dehydrogenation, which is beneficial for the generation of the target product C_3_H_6_ [34]. However, in the third dehydrogenation, the Pt_1_ site still gives C_3_H_6_ a small dehydrogenation energy barrier. This leads to a small amount of C_3_H_6_ deeply cracking into CH_4_ and C_2_H_6_. Since the literature only provides theoretical calculations, the selectivity of the Pt_1_ site in specific experiments is not clear, so further research is needed. It should be pointed out that in catalysts with a high Sn/Pt ratio, this type of adsorption was limited. Therefore, doping Pt with an appropriate amount of Sn can dilute the Pt atoms in the particles, resulting in continuous Pt clusters to segregate into isolated Pt atoms.

### 2.3. Influence of Acid Impregnation on Catalyst Performance

**Figure 5:**
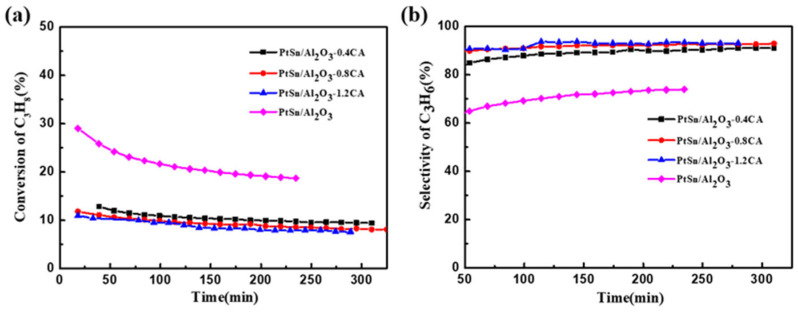
The catalytic performance of PtSn/Al_2_O_3_ catalysts with different acidity for the propane dehydrogenation reaction: (**a**) conversion; (**b**) selectivity (reaction conditions: PtSn/Al_2_O_3_ 100 mg; WHSV(C_3_H_8_) = 4.7 h^−1^; T = 580 °C; C_3_H_8_:N_2_ = 1:5).

**Figure 6:**
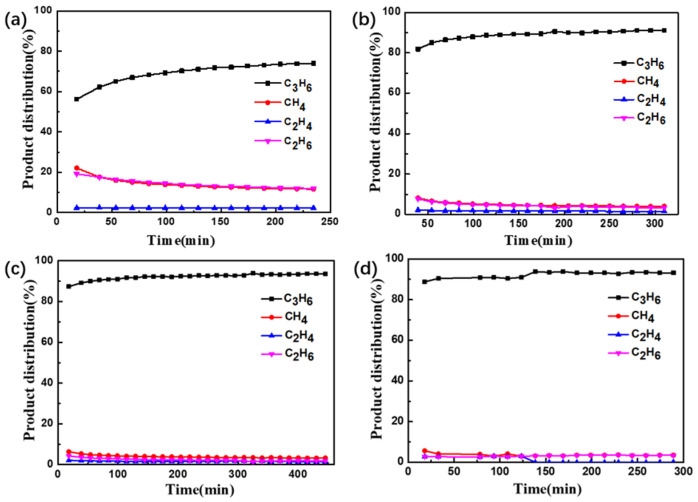
Products of propane dehydrogenation rection by PtSn/Al_2_O_3_ catalyst: (**a**) PtSn/Al_2_O_3_, (**b**) PtSn/Al_2_O_3_-0.4CA, (**c**) PtSn/Al_2_O_3_-0.8CA, (**d**) PtSn/Al_2_O_3_-1.2CA.

By altering the acidity of the catalyst, the effects of acidity on the catalytic performance of PDH were investigated. Figure 5 illustrated the catalytic performance of PtSn/Al_2_O_3_ catalysts with different acidity levels in the PDH reaction. The catalytic performance of the PtSn/Al_2_O_3_ catalyst significantly decreased in the first 20 min of the PDH reaction. After about 4 h of reaction, the final conversion is 18.7%. At the same reaction temperature and WHSV, the PtSn/Al_2_O_3_ catalyst efficiency was about 10% after 4 h of the reaction [8]. In contrast, the PtSn/Al_2_O_3_ catalyst treated with citric acid competition impregnation exhibited a lower propane conversion compared to the untreated PtSn/Al_2_O_3_ catalyst. However, the deactivation constant of PtSn/Al_2_O_3_-CA series catalysts was lower (Table 2), indicating better stability. This may be due to the increased immersion depth of the active metal Pt in the catalyst prepared using citric acid impregnation. For the commercial PDH catalyst Pt/Al_2_O_3_, the active metal is loaded on the surface of the support. During the PDH fluidized bed reaction, commercial catalysts inevitably wear out and deactivate. In this study, Pt and Sn were distributed both on the surface and in the deeper layers of the catalyst. This design avoided catalyst deactivation caused by surface Pt metal loss, maintaining a stable conversion. In addition, compared with untreated PtSn catalysts, the selectivity of catalysts impregnated with citric acid was greatly improved, which was precisely due to the increase in Pt_1_ sites during the impregnation process. The products of the PDH reaction process were shown in Figure 6. Obviously, the fresh PtSn/Al_2_O_3_ catalyst yielded the most abundant by-products, with methane and ethylene as the main components. This further suggested that strong acid sites promote the cleavage of C–C bonds during the reaction. In contrast, PtSn/Al_2_O_3_-CA catalysts primarily produce propylene, with low proportions of methane and ethane as by-products. Notably, the PtSn/Al_2_O_3_-1.2AC catalyst shows no detectable ethylene content in by-products after 150 min of the PDH reaction.

**Figure 7:**
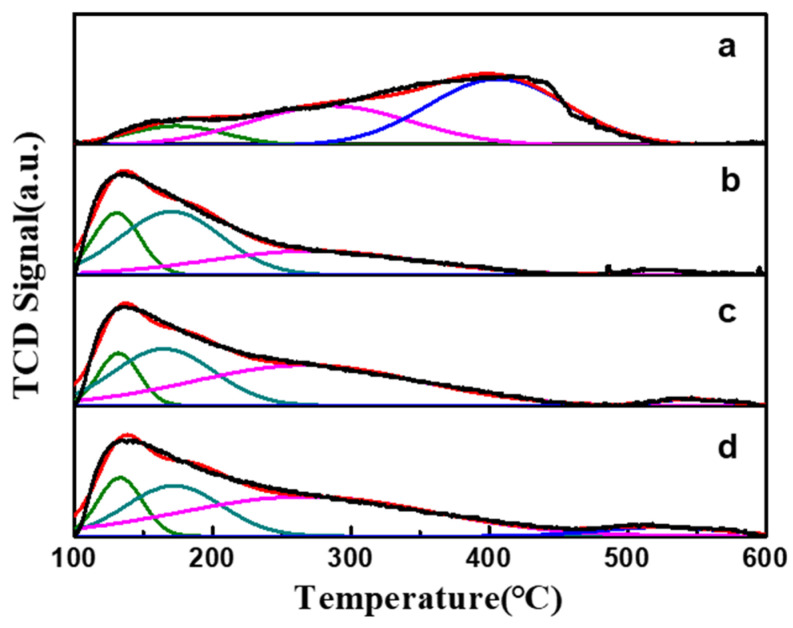
NH_3_-TPD spectra of PtSn/Al_2_O_3_ and competitive adsorbents with citric acid-added samples: (**a**) PtSn/Al_2_O_3_, (**b**) PtSn/Al_2_O_3_-0.4CA, (**c**) PtSn/Al_2_O_3_-0.8CA, (**d**) PtSn/Al_2_O_3_-1.2CA.

To elucidate this phenomenon, the acid sites of the PtSn/Al_2_O_3_ catalyst were further analyzed. The NH_3_-TPD curves of the fresh PtSn/Al_2_O_3_ catalyst showed three desorption peaks with maximum temperatures of 173 °C, 286 °C, and 406 °C, respectively (Figure 7a). After impregnation with citric acid, four more desorption peaks were observed. NH_3_ desorption in temperature ranges of 120~200 °C, 200~350 °C, and 350 °C corresponds to weak, medium, and strong acid sites, respectively. To obtain semi-quantitative results for total acidity and acid strength distribution, a Gaussian peak fitting method was used to deconvolute the NH_3_-TPD curves. The fitted peaks and results were shown in Table 3. The total peak areas for PtSn/Al_2_O_3_-0.4CA, PtSn/Al_2_O_3_-0.8CA, and PtSn/Al_2_O_3_-1.2CA catalysts are 407.9, 480.4, and 515.9, respectively. From previous studies, side reactions, such as cracking, isomerization, and coking, were mainly due to strong acid centers [35]. With the increase in citric acid content, the number of strong acid centers significantly decreased, while the number of weak acid centers increased. Therefore, the selectivity and stability of catalysts were improved.

**Figure 8:**
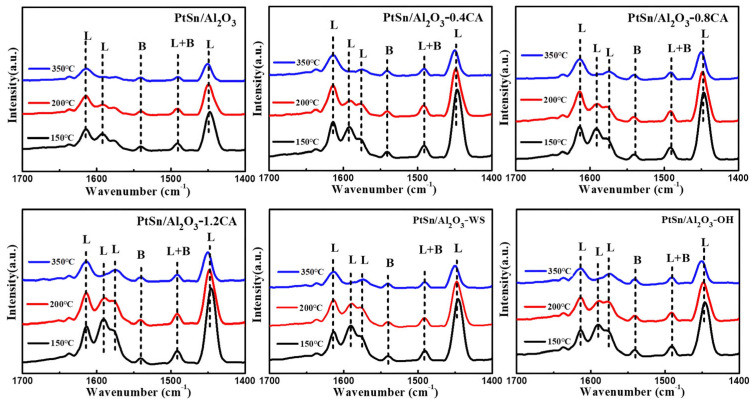
Pyridine Infrared Spectroscopy of PtSn/Al_2_O_3_ series catalysts.

By using pyridine as a probe molecule, the types of acids were further characterized. Figure 8 demonstrated the acid sites of the catalyst at desorption temperatures of 150 °C, 200 °C, and 300 °C, and the acid amounts were quantitatively analyzed (shown in Table S3). The adsorption peaks around 1450 cm^−1^ and 1600 cm^−1^ were usually associated with pyridine adsorption on Lewis acid sites [36], while the peak at 1541 cm^−1^ was related to pyridine adsorption on Brønsted acid sites [36]. The peak at 1490 cm^−1^ was attributed to the interaction of pyridine with both Lewis and Brønsted acid sites [36], and the intensity of the peaks significantly diminishes with higher temperatures [37]. From Figure 8, the acid site location was essentially the same before and after the addition of citric acid. However, the Lewis acid sites of PtSn/Al_2_O_3_-CA were enhanced after the addition of citric acid (shown in Table S3). The stronger Lewis acid sites were prone to induce coking, which may be a reason for the decreased conversion of the PtSn/Al_2_O_3_-CA catalyst.

Based on PtSn/Al_2_O_3_-0.4CA, mild alkali neutralization and water washing catalysts were further performed to investigate the catalysts’ acidity (Figure S7). PtSn/Al_2_O_3_-WS and PtSn/Al_2_O_3_-OH catalysts maintained the selectivity of over 96% after nearly 4 h of the PDH reaction. Compared to PtSn/Al_2_O_3_-CA, the selectivity further increased. Similarly, PtSn/Al_2_O_3_-WS, PtSn/Al_2_O_3_-OH, and PtSn/Al_2_O_3_ exhibit similar Brønsted acid sites and Lewis acid sites (Figure S6). However, due to the further decrease in the strength of its strong Lewis acid sites, its propylene selectivity was further improved.

XPS was used to investigate the effects of the chemical state of active metals in the catalyst on its catalytic activity. For PtSn/Al_2_O_3_-WS and PtSn/Al_2_O_3_-OH catalysts, the binding energies of Pt^0^, Pt^2+^, and Pt^4+^ all shifted to lower energy levels. It indicated an electron-rich Pt state (Figure S8 and Table S5). For the PtSn/Al_2_O_3_ catalyst, the metallic Pt content was 37.5%, which increased to 45.2% after alkali neutralization. The PtSn/Al_2_O_3_-WS catalyst showed a further increase in the content of metallic Pt to 58.3%, which was more favorable for the selectivity of the catalyst [38].

### 2.4. Reaction Thermodynamic Analysis of Catalyst

**Figure 9:**
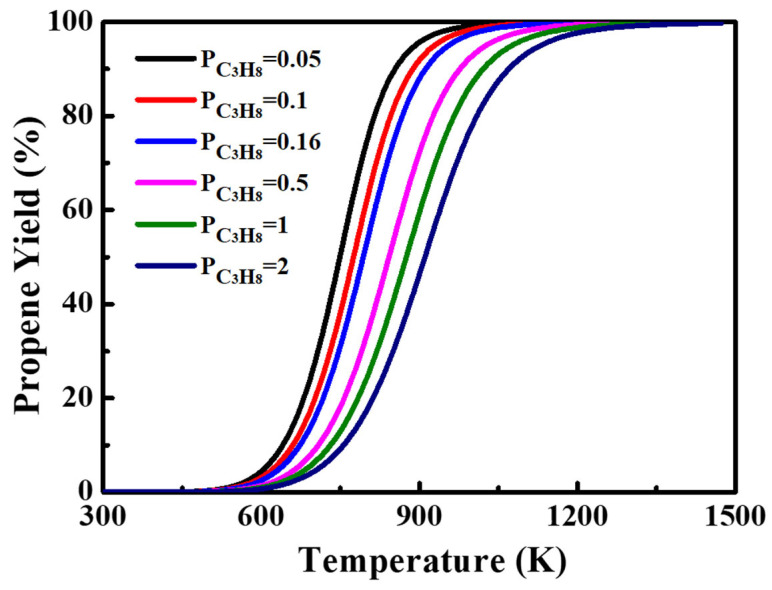
The pressure dependence of propane as a function of temperature.

A detailed thermodynamic analysis was conducted as shown in Figure 9. The propane dehydrogenation reaction was significantly influenced by the component pressure. The propane dehydrogenation reaction was conducted at 580 °C in this study, where the thermodynamic limit of the propane conversion ratio was around 30%. To further increase the utilization ratio of propane, it is suggested to conduct the reaction at a lower propane partial pressure at the thermodynamic viewpoint, but it would be economically unfavored if too low of a propane partial pressure is applied.

**Figure 10:**
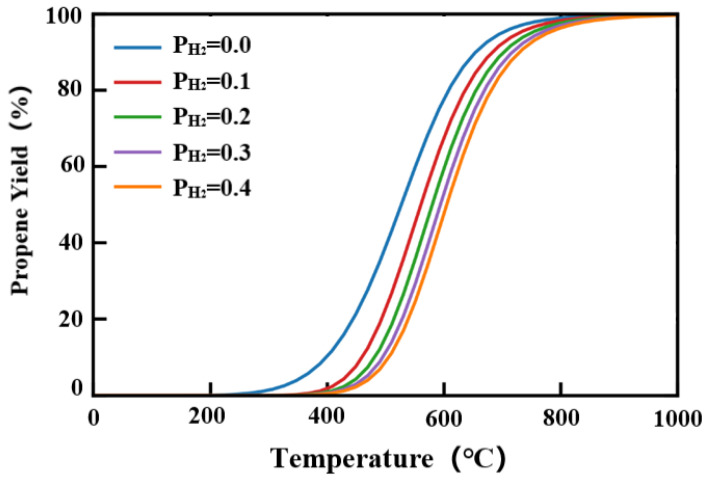
The pressure dependence of hydrogen as a function of temperature.

The propene yield is also dependent on hydrogen partial pressure, as shown in Figure 10. Hydrogen is a by-product of the propane dehydrogenation reaction, which also reversely suppresses the reaction. Theoretically, if hydrogen could be totally removed in the system, propene could reach a yield limit of up to around 60% at 580 °C. The working condition of the catalyst applied in this study only limited the propene yield to around 30%. A lower hydrogen partial pressure would favor the yield of propene, but the possible deactivation due to carbon deposition should also be considered.

**Figure 11:**
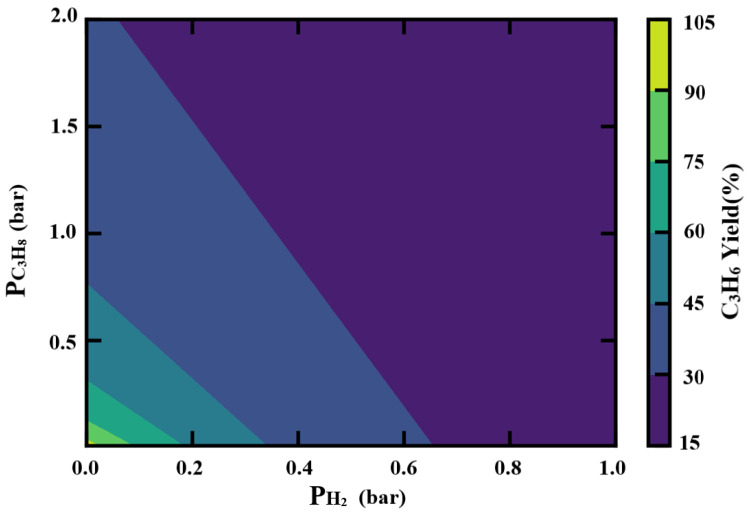
Pressure dependence on the combination effects of hydrogen and propane.

By combining the dependencies of two components in the reaction, the yield of propene can be calculated as shown in Figure 11. The yield of propene favors a condition where both hydrogen and propane are in relatively low pressure. But the contour line has a deeper slope, which means the drop in hydrogen pressure would contribute more compared with propane pressure. Herein, the optimal working condition would be a relatively sub-atmospheric pressure environment, with a lower propane pressure. Hydrogen should be removed as much as possible when recycling the reaction gas to ensure a higher propane utilization ratio.

## 3. Discussion

A PtSn bimetallic catalyst supported on γ-Al_2_O_3_ was prepared using citric acid as a competitive adsorbent, and a series of characterizations were carried out. No diffraction peaks of Pt and Sn were detected in the XRD spectrum, indicating a good dispersion of Pt and Sn on the alumina support. By using citric acid as a competitive adsorbent to regulate the immersion depth of active metals Pt and Sn, the apparent concentration and atomic percentage of metal Pt increase after adding citric acid, with the PtSn/Al_2_O_3_-0.4CA catalyst being the highest. However, as the citric acid content increases, the apparent concentration and atomic percentage of Pt begin to decrease, which may be due to excessive citric acid covering the active metal Pt. XPS shows that for the PtSn/Al_2_O_3_-CA catalyst, the binding energies of Pt^0^ and Pt^2+^ shift towards lower energy levels with the addition of CA, indicating that Pt tends to be more electron rich. The characterization of acid sites revealed that the addition of citric acid regulated the distribution of acid sites in the catalyst, and the number of strong acid sites significantly decreased. The catalytic performance of the catalyst was further evaluated with GC-MS, and it was found that compared with the untreated PtSn/Al_2_O_3_ catalyst, the selectivity of the PtSn/Al_2_O_3_-CA catalyst was greatly improved, which is attributed to the increase in Pt^0^ sites during the impregnation process. With the addition of citric acid, the number of strong acid sites in PtSn/Al_2_O_3_-CA catalysts significantly decreases, the number of weak acid sites increases, and the side reactions caused by strong acid sites decrease, which is beneficial for improving selectivity and stability. In addition, thermodynamic and kinetic analyses show that under appropriate conditions, lower hydrogen and propane pressures are beneficial for obtaining higher propylene yields, and compared to propane pressure, a decrease in hydrogen pressure is more conducive to improving the yield. In the future, in-depth research on the impact of changes in catalyst acidity and alkalinity on catalytic performance will help optimize the reaction conditions of propane dehydrogenation to propylene technology and improve propylene yield and selectivity.

## 4. Materials and Methods

### 4.1. Synthesis of Catalysts

Chemicals used in this study included chloroplatinic acid (H_2_PtCl_6_.6H_2_O > 37.5% Pt AR, Aladdin, Bay City, MI, USA), stannous oxalate (SnC_2_O_4_ AR, Aladdin), sasol boehmite (SB) powder (AR, Aladdin), anhydrous calcium chloride (CaCl_2_ AR, Aladdin), sodium alginate ((C_6_H_7_O_6_Na)n AR, Aladdin), citric acid monohydrate (CA, C_6_H_8_O_7_·H_2_O AR, Aladdin), potassium hydroxide (KOH AR, Shanghai McLean, Shanghai, China), and hydrochloric acid (HCl AR, Sinopharm, Beijing, China). Gases used in PDH testing included propane (16% C_3_H_8_, Shougang Gases, Beijing, China), hydrogen (20% H_2_, Shougang company, Beijing, China), and nitrogen (20% N_2_, Shougang Gases). All the above materials were used without further processing.

Preparation of Sn/Al_2_O_3_ pellets: CaCl_2_ (4.0000 g) was dissolved in 400 mL water, then hydrochloric acid was added to adjust the solution pH to 2. We mixed 20.000 g of SB powder with SnC_2_O_4_ (0.3297 g), took 3.750 g of the mixed powder and stirred it with 10 mL water, and then added 0.090 g of sodium alginate to form a slurry. It was stirred for 2 h until all material mixed evenly. The above slurry was injected into a syringe and dripped into the calcium chloride solution to form small pellets. After 30 min of reaction, the pellets were cleaned with pure water 3 times and dried in ambient air completely. The pellets were calcined in a muffle furnace with the following process: calcined 30 min from room temperature to 110 °C, kept for 30 min; calcined 60 min from 110 °C to 350 °C, kept for 30 min; and calcined 120 min from 350 °C to 560 °C, kept for 4 h. The Sn/Al_2_O_3_ pellets were prepared after calcination.

Synthesis of PtSn/Al_2_O_3_ catalyst: The Sn/Al_2_O_3_ pellets (3.0000 g) were soaked in 30 mmol/L chloroplatinic acid solution (3.3 mL) for 3 h. After impregnation, the sample was dried overnight at 110 °C and calcined for 2 h at 400 °C to obtain PtSn/Al_2_O_3_ catalyst.

Synthesis of PtSn/Al_2_O_3_-CA series catalysts: We mixed 3.3 mL chloroplatinic acid (30 mmol/L) with 0.1680 g CA to prepare an impregnation solution. The Sn/Al_2_O_3_ pellets (3.0000 g) were soaked into the impregnation solution for 3 h. Then, the sample was dried overnight at 110 °C and calcined in a muffle furnace at 400 °C for 2 h to obtain PtSn/Al_2_O_3_-0.4CA. Following the above steps, only changing the amount of citric acid added to the impregnation solution to 0.3360 g and 0.5040 g resulted in PtSn/Al_2_O_3_-0.8CA and PtSn/Al_2_O_3_-1.2CA catalysts with citric acid concentrations of 0.8 mol/L and 1.2 mol/L, respectively.

Synthesis of PtSn/Al_2_O_3_-WS and PtSn/Al_2_O_3_-OH: The PtSn/Al_2_O_3_ catalyst pellets (3.0000 g) were soaked in water and potassium hydroxide (0.35 mol/L) solution for 60 min, respectively. The catalysts were then cleaned with deionized water 3 times and then dried in a 50 °C oven overnight to obtain PtSn/Al_2_O_3_-WS and PtSn/Al_2_O_3_-OH catalysts, respectively.

### 4.2. Characterization of Catalysts

The characterization of catalyst morphology was performed on a field emission scanning electron microscope instrument (SEM JMS-7500F, Nippon Electronics, Tokyo, Japan). The instrument is equipped with an 80 mm^2^ energy-dispersive X-ray spectrometer (EDS, Oxford, UK). The specific surface area and pore volume of the samples were analyzed with a nitrogen adsorption instrument (NADS, ASAP 2046M, Micromeritics, Norcross, GA, USA) through N_2_ adsorption/desorption isotherms under 77 K. The specific surface area of the catalysts was calculated using the Brunauer–Emmett–Teller (BET) method, and the pore volume and pore size were determined using the Barrett–Joyner–Halenda (BJH) method. An X-ray diffractometer (XRD, 18KW D/MAX2500V+/PC, Rigaku Corporation, Akishima, Japan) was used to determine the surface chemical states of samples. The electronic states of the metal and the surface composition of the catalyst were determined through photoelectron spectroscopy (XPS ESCALAB250, Thermo Fisher Scientific, Waltham, MA, USA).

The infrared spectrum of CO as the probe was measured using a Fourier transform infrared spectrometer (FTIR, Thermo Fisher Scientific). About 50 mg samples were reduced at 540 °C pure H_2_ (20 mL/min) for 60 min and then cooled at pure N_2_ (20 mL/min) to 30 °C to collect background spectra. CO (20 mL/min) was injected for 30 min, and then, N_2_ (20 mL/min) was purged individually and purified for 30 min during experiments.

The acid sites on the catalyst surface were determined with programmed temperature desorption method (NH_3_-TPD AutoChem1 II 2920, microstructures). Approximately 50 mg of the sample was pretreated at 300 °C in He (30 mL/min) for 60 min to remove moisture, physically adsorbed water, and other impurities. After pretreatment, 5% NH_3_ (30 mL/min, balanced with He) was introduced at 50 °C for 60 min, followed by He gas (30 mL/min) purging at 50 °C for 60 min. The TPD data were recorded as the temperature range from 50 to 800 °C under He gas.

The type of acid was determined with pyridine as the probe molecule and with in situ diffuse reflectance infrared spectroscopy (DRIFT Nicolet iS50, Thermo Fisher, Waltham, MA, USA). The samples were compressed into thin sheets and placed in the reaction cell. The system was evacuated to 10^−3^ Pa at 300 °C and maintained for 60 min, followed by cooling to room temperature. Pyridine vapor was introduced into the system for 30 min until it reached equilibrium. The temperature was then raised to 200 °C, followed by another evacuation to 10^−3^ Pa, and it was kept 30 min before cooling to room temperature. Infrared spectra were scanned in the wavenumber range of 1400 to 1700 cm^−1^, and the infrared spectrum of pyridine adsorption at 200 °C was recorded. The same procedure was repeated for desorption treatments at other specified temperatures, with corresponding spectra collected.

### 4.3. Catalytic Performance Test

In a fixed-bed quartz reactor with an inner diameter of 6 mm, propane dehydrogenation on PtSn/Al_2_O_3_ catalyst was conducted under atmospheric pressure. The PDH reaction was performed in a mixture of 16 vol% C_3_H_8_, 20 vol% H_2_ and N_2_, with a propane weight hourly space velocity (WHSV) of 4.7 h^−1^ and a temperature of 580 °C. The reaction products were analyzed using gas chromatography with a flame ionization detector (FID). FID was employed to measure the concentrations of all hydrocarbons, including CH_4_, C_2_H_6_, C_2_H_4_, C_3_H_6_, and C_3_H_8_ (with no detection of dimerization or aromatization products). The formulas for propane conversion and propylene selectivity are as follows:

(1) $${Conversion}_{\left(C_{3}H_{8}\right)}=\frac{3\times C_{\left(C_{3}H_{6}\right)}+2\times C_{\left(C_{2}H_{6}\right)}+2\times C_{\left(C_{2}H_{4}\right)}+C_{\left(CH_{4}\right)}}{3\times C_{\left(C_{3}H_{8}\right)}+3\times C_{\left(C_{3}H_{6}\right)}+2\times C_{\left(C_{2}H_{6}\right)}+2\times C_{\left(C_{2}H_{4}\right)}+C_{\left(CH_{4}\right)}}$$

(2) $${Selectivity}_{\left(C_{3}H_{6}\right)}=\frac{3\times C_{\left(C_{3}H_{6}\right)}}{3\times C_{\left(C_{3}H_{6}\right)}+2\times C_{\left(C_{2}H_{6}\right)}+2\times C_{\left(C_{2}H_{4}\right)}+C_{\left(CH_{4}\right)}}$$

In the formulas, CH_4_, C_2_H_6_, C_2_H_4_, C_3_H_6_, and C_3_H_8_ represent the concentrations of the corresponding gas components in the outlet gas.

The stability of the PtSn/Al_2_O_3_ catalyst was quantitatively determined using the deactivation rate constant, considering deactivation as a first-order process. The deactivation rate constant is defined as follows:

(3) $$k_{d}=\frac{1}{t}\left(\ln{\left(\frac{1-Conv.\left(C_{3}H_{8}\right)}{Conv.\left(C_{3}H_{8}\right)}\right)}_{final}-\ln{\left(\frac{1-Conv.\left(C_{3}H_{8}\right)}{Conv.\left(C_{3}H_{8}\right)}\right)}_{initial}\right)$$

### 4.4. Reaction Equilibrium Calculation

The reaction equilibrium is calculated based on van’t Hoff equation:

(4) $$\frac{d}{dT}{lnK}_{eq}=\frac{{∆}_{r}H^{{}^{\circ}}}{{RT}^{2}}$$

where *K*_eq_ is the equilibrium constant at a given temperature *T*; Δ_r_*H*° is the standard reaction enthalpy change, which can be calculated from reference values; and *R* is the ideal gas constant.

The standard equilibrium constant *K*°_eq_ can be calculated by its relationship with Gibbs free energy Δ_r_*G*°, which can also be calculated from reference values:

(5) $$K_{eq}^{{}^{\circ}}=-RT\;ln{∆}_{r}G^{{}^{\circ}}$$

For the propane dehydrogenation reaction, the reaction equilibrium constant can be used to calculate the theoretical reaction limit:

(6) $$K_{eq}=\frac{[C_{3}H_{6}{]}_{out}\;\cdot [H_{2}{]}_{out}}{[C_{3}H_{8}{]}_{out}}$$

where the notation “out” stands for the outlet concentration.

By applying the law of mass conservation, the outlet concentration can be related to the inlet or initial concentration. With tedious calculation, the outlet propene concentration can be calculated as follows:

(7) $$[C_{3}H_{6}{]}_{out}=\frac{[C_{3}H_{6}{]}_{in}-[H_{2}{]}_{in}-K_{eq}}{2}+\frac{\sqrt{[C_{3}H_{6}{]}_{in}^{2}-2[C_{3}H_{6}{]}_{in}[H_{2}{]}_{in}+2[C_{3}H_{6}{]}_{in}K_{eq}+4[C_{3}H_{8}{]}_{in}K_{eq}+([H_{2}{]}_{in}+K_{eq}{)}^{2}}}{2}$$

where the notation “in” stands for inlet or initial concentration.

## 5. Conclusions

This section is not mandatory but can be added to the manuscript if the discussion is unusually long or complex.

In this study, we used citric acid as a competing adsorbent to prepare a PtSn bimetallic catalyst supported on γ-Al_2_O_3_ and conducted a series of characterizations to study the effects of acid to the PDH reaction. The conclusions are as follows:

(1) Through characterization analyses, such as EDS, XRD, and N_2_ adsorption–desorption, Pt and Sn active metals are well dispersed. SEM scanning showed that the addition of an appropriate amount of citric acid increased the apparent concentration and immersion depth of the active metal Pt. Citric acid, as a competitive adsorbent, increased the number of Pt1 sites during the impregnation process.
(2) With the effects of acid sites, compared to a fresh PtSn/Al_2_O_3_ catalyst, the addition of citric acid led to a slight decrease in PDH conversion. However, the selectivity of the catalyst increased significantly from 74% to 93%. After neutralization with alkali and washing treatment, the selectivity of the catalyst further improved to 96%.
(3) The fresh PtSn/Al_2_O_3_ catalyst possesses the strongest acid sites. During the PDH reaction, the strong acid sites promote the cleavage of C–C bonds, leading to the generation of more by-products, such as methane and ethylene. This, in turn, reduces the selectivity of the catalyst.
(4) From the PDH reaction thermodynamic analysis of catalyst, a relatively sub-atmospheric pressure environment with a lower propane pressure could be the reasonable choice. When recovering reaction gases, hydrogen should be removed as much as possible to ensure higher propane utilization efficiency.

## Figures and Tables

**Table 1:** Peaks of the Pt and Sn species of the PtSn/Al_2_O_3_ catalysts.

Sample	Binding Energy (eV)					
	Pt 4d_5/2_			Pt 4d_3/2_		
	Pt^0^	Pt^2+^	Pt^4+^	Pt^0^	Pt^2+^	Pt^4+^
PtSn/Al_2_O_3_	309.5	316.9	320.1	332.0	334.2	336.4
PtSn/Al_2_O_3_-0.4CA	306.9	314.0	319.7	331.4	334.1	337.2
PtSn/Al_2_O_3_-0.8CA	306.7	314.6	323.7	330.3	333.4	337.1
PtSn/Al_2_O_3_-1.2CA	307.4	314.9	321.0	329.4	334.1	339.2
**Sample**	**Sn 4d_5/2_**			**Sn 4d_3/2_**		
	**Sn^0^**	**Sn^2+^**	**Sn^4+^**	**Sn^0^**	**Sn^2+^**	**Sn^4+^**
Sn/Al_2_O_3_	495.1	495.8	496.5	486.7	487.5	488.2

**Table 2:** The catalytic performance of series catalysts PtSn/Al_2_O_3_ for PDH reaction.

Sample	Conversion (%)		Selectivity (%)		K _d_^a^ (h^−1^)
	Initial	Final	Initial	Final	
PtSn/Al_2_O_3_	29.0	18.7	56.2	74.0	0.0024
PtSn/Al_2_O_3_-0.4 CA	12.9	9.4	81.8	91.0	0.0011
PtSn/Al_2_O_3_-0.8 CA	11.8	8.1	87.4	93.9	0.0013
PtSn/Al_2_O_3_-1.2 CA	11.0	7.6	88.7	93.1	0.0014

^a^ Deactivation rate constant.

**Table 3:** NH_3_-TPD fitting results of PtSn/Al_2_O_3_ catalyst.

Sample	T_M_ (°C)				Peak Area (a.u.)			Total Area (a.u.)
	Weak		Medium	Strong	Weak	Medium	Strong	
PtSn/Al_2_O_3_	173		286	406	52.7	176.6	256.9	486.2
PtSn/Al_2_O_3_-0.4CA	131	170	273	523	258.2	140.1	9.6	407.9
PtSn/Al_2_O_3_-0.8CA	132	165	268	547	215.8	252.3	12.3	480.4
PtSn/Al_2_O_3_-1.2CA	133	172	268	524	206.3	278.9	30.7	515.9

## Data Availability

Data are contained within the article and Supplementary Materials.

## Supplementary Materials

The following supporting information can be downloaded at: https://www.mdpi.com/article/10.3390/molecules29132959/s1, Figure S1: SEM images of γ-Al_2_O_3_ support; Figure S2: SEM images of PtSn/Al_2_O_3_ catalyst; Figure S3: EDS of PtSn/Al_2_O_3_ catalyst: (a) Pt element scanning; (b) gray value of line from EDS; Figure S4: N_2_ adsorption isotherms and pore size distribution of PtSn bimetallic catalyst; Figure S5: Infrared spectra of pyridine at 200 °C before and after adding citric acid; Figure S6: Pyridine infrared spectra of catalysts treated with alkali neutralization and water washing; Figure S7: The catalytic performance of PtSn/Al_2_O_3_ catalyst treated with alkali neutralization and water washing: (a) conversion, (b) selectivity; Figure S8: XPS spectra of PtSn/Al_2_O_3_ catalyst: (a) PtSn/Al_2_O_3_, (b) PtSn/Al_2_O_3_-WS, (c) PtSn/Al_2_O_3_-OH; Table S1: BET specific surface area, pore volume, and average pore size of PtSn bimetallic catalysts and supports; Table S2: SEM line-scan elemental analysis of PtSn/Al_2_O_3_ catalyst; Table S3: Pyridine infrared quantitative results (unit: μmol/g); Table S4: The catalytic performance of PtSn/Al_2_O_3_ catalyst for the propane dehydrogenation reaction; Table S5: The peak centers of the Pt species of the PtSn/Al_2_O_3_ catalysts.
