# Supplementary material for: The Acid Roles of PtSn@Al2O3 in the Synthesis and Performance of Propane Dehydrogenation
Source: Molecules. 2024 Jun 21;29(13):2959. doi: 10.3390/molecules29132959 (PMC11243666; doi:10.3390/molecules29132959)
Supplement: Supplementary file 1 [file molecules-29-02959-s001.zip › molecules-3001688-supplementary.pdf]

**Supporting Information**

# The Acids roles of PtSn@$Al_2O_3$ in the Synthesis and Performance of Propane Dehydrogenation

Hejingying Niu[1], Jinhua Ma[1], Lina Gan[2,*], Kezhi Li[3,*]

[1] *School of Environmental & Chemical Engineering, Shanghai University, Shanghai 200444, China*
[2] *School of Environment and Architecture, University of Shanghai for Science and Technology, Shanghai 200093, China*
[3] *Institute of Engineering Technology, Sinopec Catalyst Co., Ltd., Beijing 101111, China*

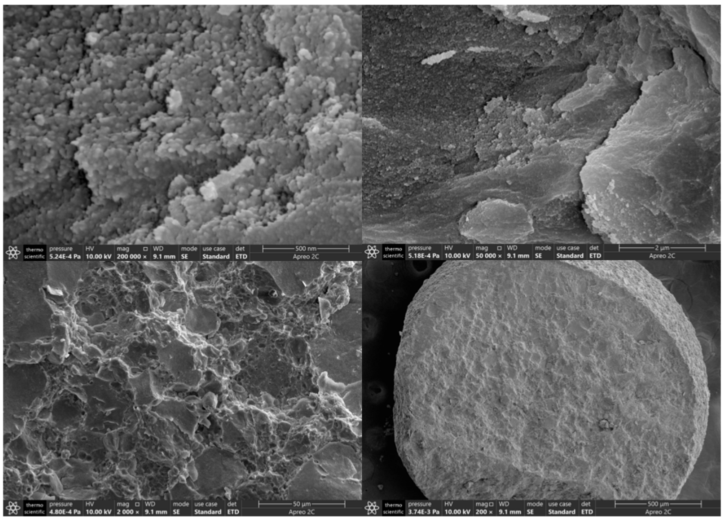

**Figure S1** SEM images of $\gamma$-$Al_2O_3$ support

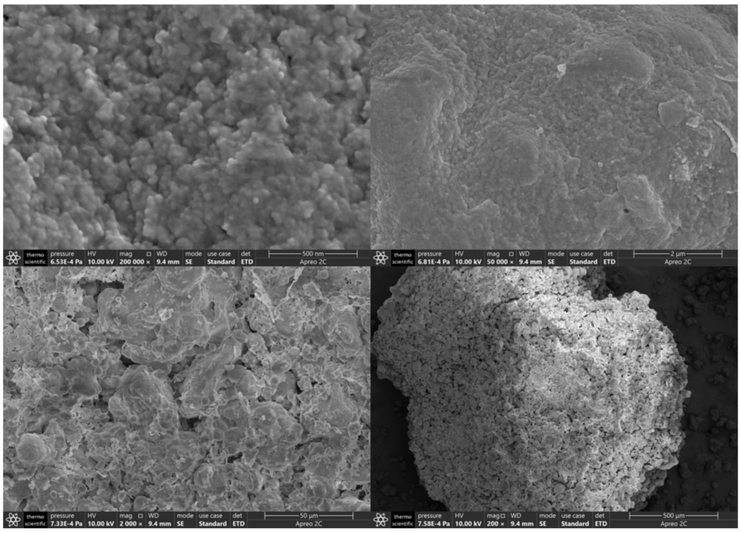

**Figure S2** SEM images of PtSn/$Al_2O_3$ catalyst

* Corresponding authors.
*E-mail addresses*: lngan@usst.edu.cn (LN. Gan), likzh.chji@sinopec.com (KZ, Li)

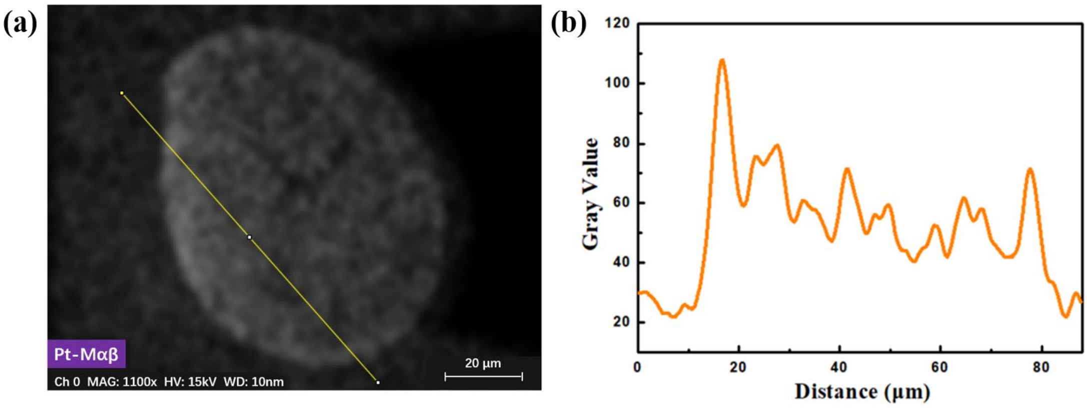


**Figure S3** EDS of PtSn/$Al_2O_3$ catalyst (a) Pt element scanning; (b) gray value of line from EDS

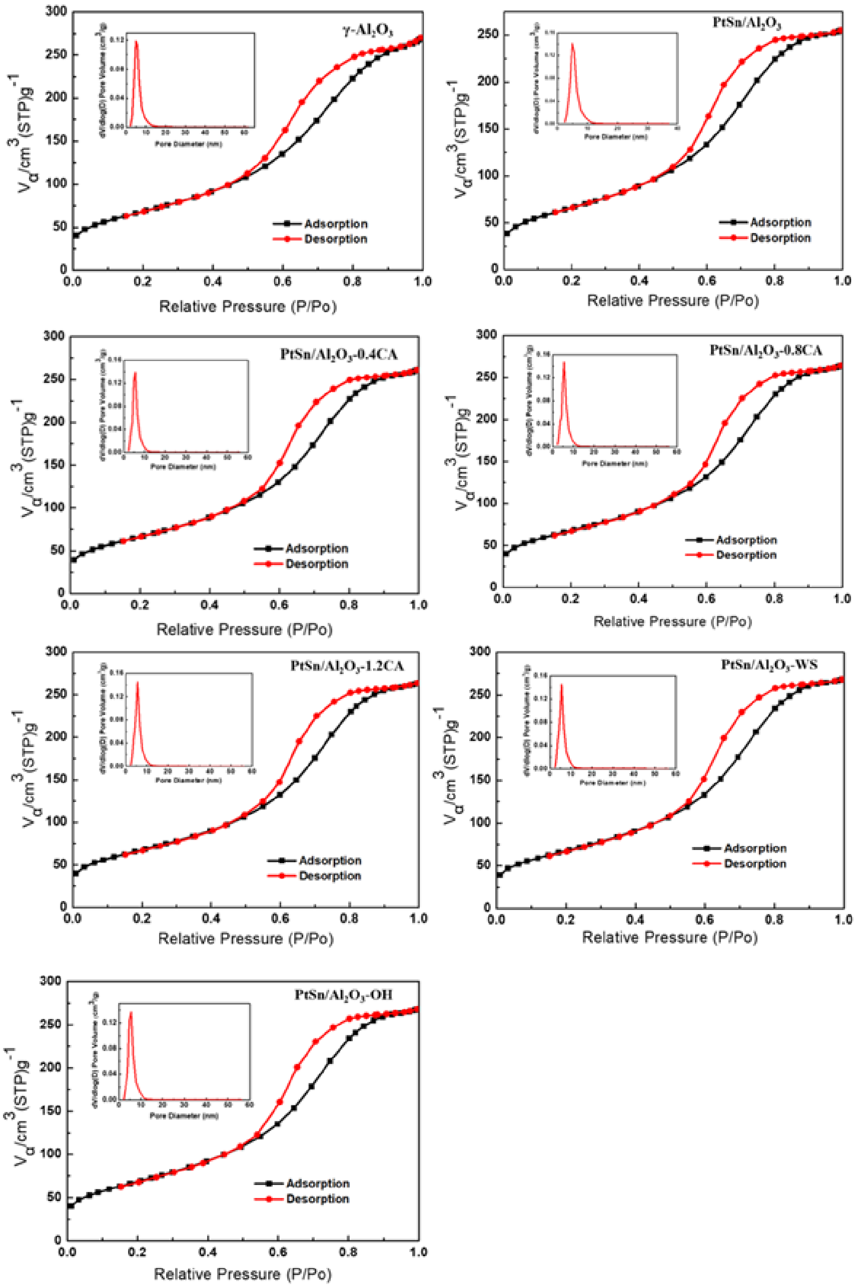


**Figure S4** $N_2$ adsorption isotherms and pore size distribution of PtSn bimetallic catalyst

**Table S1** BET specific surface area, pore volume, and average pore size of PtSn bimetallic

catalysts and supports

| Sample | BET area ($m^2g^{-1}$) | Pore volume ($cm^3g^{-1}$) | Pore size (nm) |
|---|---|---|---|
| γ-$Al_2O_3$ | 250 | 0.44 | 5.6 |
| PtSn/$Al_2O_3$ | 241 | 0.42 | 5.4 |
| PtSn/$Al_2O_3$-0.4CA | 241 | 0.43 | 5.6 |
| PtSn/$Al_2O_3$-0.8CA | 245 | 0.43 | 5.6 |
| PtSn/$Al_2O_3$-1.2CA | 245 | 0.43 | 5.6 |
| PtSn/$Al_2O_3$-WS | 245 | 0.44 | 5.6 |
| PtSn/$Al_2O_3$-OH | 250 | 0.44 | 5.5 |

**Table S2** SEM line scan elemental analysis of PtSn/$Al_2O_3$ catalyst

| Sample | element | Line Type | apparent concentration | k ratio | wt% | wt% Sigma | Atomic percentage |
|---|---|---|---|---|---|---|---|
| PtSn/$Al_2O_3$ | Pt | M | 0.23 | 0.00230 | 3.81 | 0.34 | 0.41 |
| PtSn/$Al_2O_3$-0.4CA | Pt | M | 0.30 | 0.00303 | 3.85 | 0.33 | 0.42 |
| PtSn/$Al_2O_3$-0.8CA | Pt | M | 0.26 | 0.00259 | 3.68 | 0.39 | 0.40 |
| PtSn/$Al_2O_3$-1.2CA | Pt | M | 0.25 | 0.00247 | 3.38 | 0.27 | 0.36 |

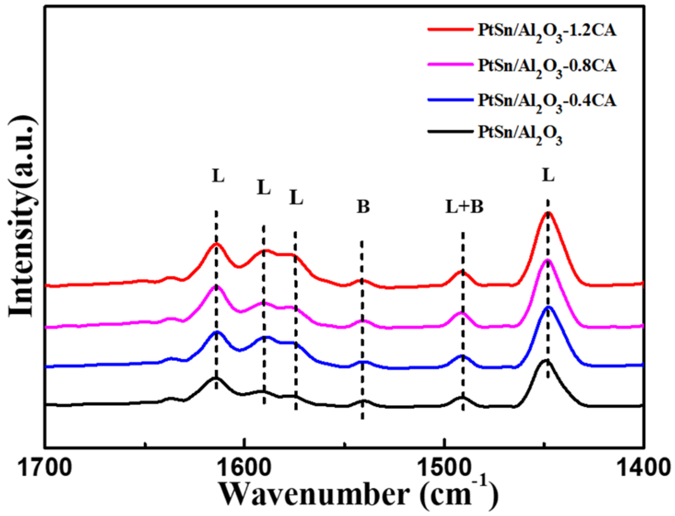


**Figure S5** Infrared spectra of pyridine at 200℃ before and after adding citric acid

**Table S3** Pyridine infrared quantitative results (unit: μmol/g)

| Sample | 150℃ | | 200℃ | | 350℃ | |
|---|---|---|---|---|---|---|
| | B | L | B | L | B | L |
| PtSn/$Al_2O_3$ | 17.55 | 211.06 | 16.04 | 161.27 | 13.19 | 78.77 |
| PtSn/$Al_2O_3$-0.4CA | 20.02 | 307.71 | 18.19 | 213.34 | 17.55 | 101.69 |
| PtSn/$Al_2O_3$-0.8CA | 19.86 | 311.37 | 18.67 | 227.86 | 17.79 | 111.77 |
| PtSn/$Al_2O_3$-1.2CA | 18.43 | 349.05 | 16.28 | 258.58 | 14.38 | 119.51 |
| PtSn/$Al_2O_3$-WS | 18.27 | 289.42 | 16.60 | 213.16 | 15.41 | 91.07 |
| PtSn/$Al_2O_3$-OH | 20.89 | 255.76 | 19.14 | 189.52 | 17.47 | 92.15 |

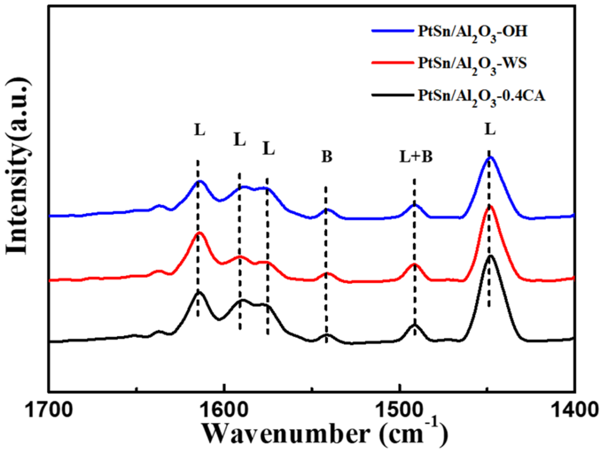


**Figure S6** Pyridine infrared spectra of catalysts treated with alkali neutralization and water washing at 200℃

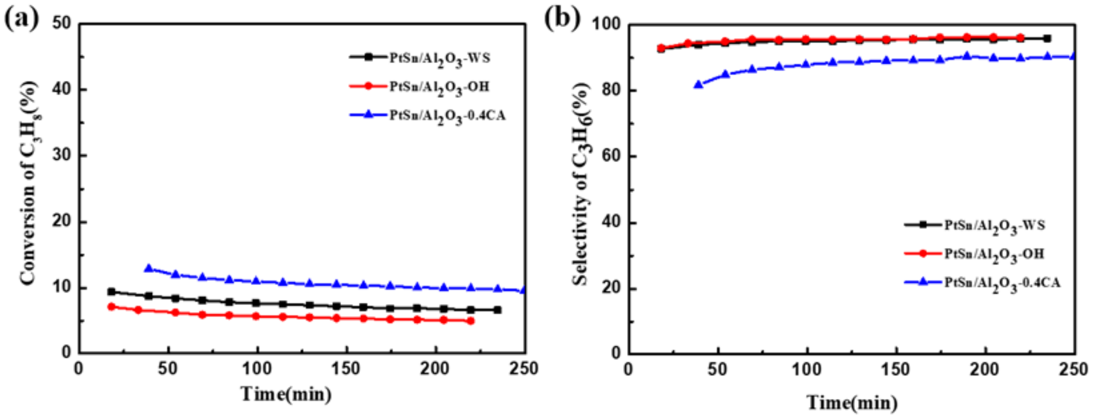


**Figure S7** The catalytic performance of PtSn/$Al_2O_3$ catalyst treated with alkali neutralization and water washing(**a**) Conversion; (**b**) Selectivity

**Table S4** The catalytic performance of PtSn/$Al_2O_3$ catalyst for propane dehydrogenation reaction

| Sample | Conversion (%) | | Selectivity (%) | | $K_d{}^a$($h^{-1}$) |
|---|---|---|---|---|---|
| | Initial | Finial | Initial | Finial | |
| PtSn/$Al_2O_3$-0.4CA | 12.9 | 9.4 | 81.8 | 91.0 | 0.0011 |
| PtSn/$Al_2O_3$-WS | 9.4 | 6.6 | 92.8 | 96.0 | 0.0016 |
| PtSn/$Al_2O_3$-OH | 7.1 | 5.0 | 93.0 | 96.2 | 0.0017 |

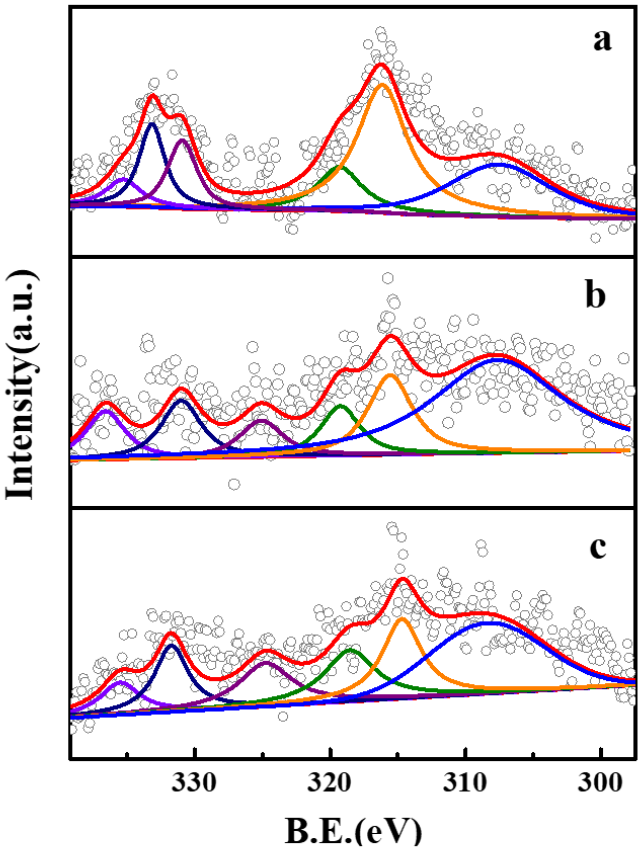


**Figure S8** XPS spectra of $PtSn/Al_2O_3$ catalyst (**a**) $PtSn/Al_2O_3$ (**b**) $PtSn/Al_2O_3$-WS (**c**) $PtSn/Al_2O_3$-OH

**Table S5** Peak centers of the Pt species of the $PtSn/Al_2O_3$ catalysts

| Sample | Binding energy(eV) | | | | | | $Pt^0/Pt^{\delta+}$ (%) |
|---|---|---|---|---|---|---|---|
| | Pt $4d_{5/2}$ | | | Pt $4d_{3/2}$ | | | |
| | $Pt^0$ | $Pt^{2+}$ | $Pt^{4+}$ | $Pt^0$ | $Pt^{2+}$ | $Pt^{4+}$ | |
| $PtSn/Al_2O_3$ | 309.5 | 316.9 | 320.1 | 332.0 | 334.2 | 336.4 | 37.5% |
| $PtSn/Al_2O_3$-WS | 307.3 | 315.0 | 318.6 | 324.3 | 330.0 | 335.5 | 58.3% |
| $PtSn/Al_2O_3$-OH | 308.4 | 314.7 | 318.5 | 324.8 | 331.7 | 355.5 | 45.2% |
